# Coronavirus Disease 2019 in Children — United States, February 12–April 2, 2020

**DOI:** 10.15585/mmwr.mm6914e4

**Published:** 2020-04-10

**Authors:** CDC COVID-19 Response Team, CDC COVID-19 Response Team, Stephanie Bialek, Ryan Gierke, Michelle Hughes, Lucy A. McNamara, Tamara Pilishvili, Tami Skoff

**Affiliations:** CDC; CDC; CDC; CDC; CDC; CDC.

*On April 6, 2020, this report was posted online as an *MMWR *Early Release.*

As of April 2, 2020, the coronavirus disease 2019 (COVID-19) pandemic has resulted in >890,000 cases and >45,000 deaths worldwide, including 239,279 cases and 5,443 deaths in the United States (*1*,*2*). In the United States, 22% of the population is made up of infants, children, and adolescents aged <18 years (children) (*3*). Data from China suggest that pediatric COVID-19 cases might be less severe than cases in adults and that children might experience different symptoms than do adults (*4*,*5*); however, disease characteristics among pediatric patients in the United States have not been described. Data from 149,760 laboratory-confirmed COVID-19 cases in the United States occurring during February 12–April 2, 2020 were analyzed. Among 149,082 (99.6%) reported cases for which age was known, 2,572 (1.7%) were among children aged <18 years. Data were available for a small proportion of patients on many important variables, including symptoms (9.4%), underlying conditions (13%), and hospitalization status (33%). Among those with available information, 73% of pediatric patients had symptoms of fever, cough, or shortness of breath compared with 93% of adults aged 18–64 years during the same period; 5.7% of all pediatric patients, or 20% of those for whom hospitalization status was known, were hospitalized, lower than the percentages hospitalized among all adults aged 18–64 years (10%) or those with known hospitalization status (33%). Three deaths were reported among the pediatric cases included in this analysis. These data support previous findings that children with COVID-19 might not have reported fever or cough as often as do adults (*4*). Whereas most COVID-19 cases in children are not severe, serious COVID-19 illness resulting in hospitalization still occurs in this age group. Social distancing and everyday preventive behaviors remain important for all age groups as patients with less serious illness and those without symptoms likely play an important role in disease transmission (*6*,*7*).

Data on COVID-19 cases were reported to CDC from 50 states, the District of Columbia, New York City, and four U.S territories. Jurisdictions voluntarily report data on laboratory-confirmed cases using a standardized case report form.* Data on cases occurring during February 12–April 2, 2020 and submitted through an electronic case-based COVID-19 surveillance database were reviewed for this report. Data submitted to CDC are preliminary and can be updated by health departments as more data become available. At the time of this analysis, characteristics of interest were available for only a minority of cases, including hospitalization status (33%), presence of preexisting underlying medical conditions (13%), and symptoms (9.4%). Because of the high percentage of cases with missing data and because cases with severe outcomes are more likely to have hospitalization or intensive care unit (ICU) status reported, percentages of patients hospitalized, including those admitted to the ICU, were estimated as a range, for which the denominator for the lower bound included cases with both known and unknown hospitalization or ICU status, and the upper bound included only cases with known hospitalization or ICU status. For other characteristics, percentages were calculated from among the number of cases with known information for that characteristic. Demographics of COVID-19 cases were assessed among cases in children aged <18 years and adults aged ≥18 years. Because clinical severity of COVID-19 is higher among adults aged ≥65 years than in younger age groups (*8*), clinical features including symptoms and hospitalizations were assessed among adults aged 18–64 years and compared with those among the pediatric cases. Statistical comparisons were not performed because of the high percentage of missing data.

As of April 2, 2020, data on 149,760 laboratory-confirmed U.S. COVID-19 cases were available for analysis. Among 149,082 (99.6%) cases for which patient age was known, 2,572 (1.7%) occurred in children aged <18 years and 146,510 (98%) in adults aged ≥18 years, including 113,985 (76%) aged 18–64 years. Among the 2,572 pediatric cases, 850 (33%) were reported from New York City; 584 (23%) from the rest of New York state; 393 (15%) from New Jersey; and the remaining 745 (29%) from other jurisdictions. The distribution of reporting jurisdictions for pediatric cases was similar to that of reporting jurisdictions for cases among adults aged ≥18 years, except that a lower percentage of adult cases was reported from New York state (14%). The first pediatric U.S. COVID-19 case was reported to CDC on March 2, 2020; since March 5, pediatric cases have been reported daily (Figure 1).

**FIGURE 1:**
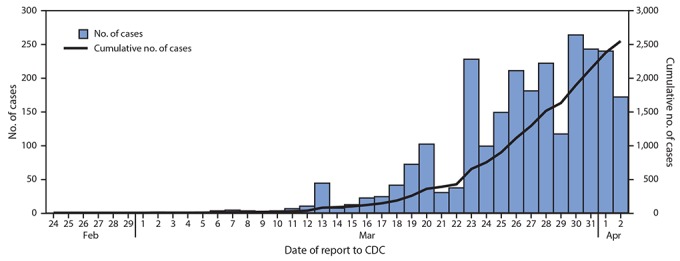
COVID-19 cases in children* aged <18 years, by date reported to CDC (N = 2,549)^†^ — United States, February 24–April 2, 2020^§^<Fig_Large></Fig_Large> * Includes infants, children, and adolescents. ^†^ Excludes 23 cases in children aged <18 years with missing report date. ^§^ Date of report available starting February 24, 2020; reported cases include any with onset on or after February 12, 2020.

Among all 2,572 COVID-19 cases in children aged <18 years, the median age was 11 years (range 0–17 years). Nearly one third of reported pediatric cases (813; 32%) occurred in children aged 15–17 years, followed by those in children aged 10–14 years (682; 27%). Among younger children, 398 (15%) occurred in children aged <1 year, 291 (11%) in children aged 1–4 years, and 388 (15%) in children aged 5–9 years. Among 2,490 pediatric COVID-19 cases for which sex was known, 1,408 (57%) occurred in males; among cases in adults aged ≥18 years for which sex was known, 53% (75,450 of 143,414) were in males. Among 184 (7.2%) cases in children aged <18 years with known exposure information, 16 (9%) were associated with travel and 168 (91%) had exposure to a COVID-19 patient in the household or community.

Data on signs and symptoms of COVID-19 were available for 291 of 2,572 (11%) pediatric cases and 10,944 of 113,985 (9.6%) cases among adults aged 18–64 years (Table). Whereas fever (subjective or documented), cough, and shortness of breath were commonly reported among adult patients aged 18–64 years (93% reported at least one of these), these signs and symptoms were less frequently reported among pediatric patients (73%). Among those with known information on each symptom, 56% of pediatric patients reported fever, 54% reported cough, and 13% reported shortness of breath, compared with 71%, 80%, and 43%, respectively, reporting these signs and symptoms among patients aged 18–64 years. Myalgia, sore throat, headache, and diarrhea were also less commonly reported by pediatric patients. Fifty-three (68%) of the 78 pediatric cases reported not to have fever, cough, or shortness of breath had no symptoms reported, but could not be classified as asymptomatic because of incomplete symptom information. One (1.3%) additional pediatric patient with a positive test result for SARS-CoV-2 was reported to be asymptomatic.

**TABLE:** Signs and symptoms among 291 pediatric (age <18 years) and 10,944 adult (age 18–64 years) patients* with laboratory-confirmed COVID-19 — United States, February 12–April 2, 2020

Sign/Symptom	No. (%) with sign/symptom	
	Pediatric	Adult
Fever, cough, or shortness of breath^†^	213 (73)	10,167 (93)
Fever^§^	163 (56)	7,794 (71)
Cough	158 (54)	8,775 (80)
Shortness of breath	39 (13)	4,674 (43)
Myalgia	66 (23)	6,713 (61)
Runny nose^¶^	21 (7.2)	757 (6.9)
Sore throat	71 (24)	3,795 (35)
Headache	81 (28)	6,335 (58)
Nausea/Vomiting	31 (11)	1,746 (16)
Abdominal pain^¶^	17 (5.8)	1,329 (12)
Diarrhea	37 (13)	3,353 (31)

*Cases were included in the denominator if they had a known symptom status for fever, cough, shortness of breath, nausea/vomiting, and diarrhea. Total number of patients by age group: <18 years (N = 2,572), 18–64 years (N = 113,985).

^†^ Includes all cases with one or more of these symptoms.

^§^ Patients were included if they had information for either measured or subjective fever variables and were considered to have a fever if “yes” was indicated for either variable.

^¶^ Runny nose and abdominal pain were less frequently completed than other symptoms; therefore, percentages with these symptoms are likely underestimates.

Information on hospitalization status was available for 745 (29%) cases in children aged <18 years and 35,061 (31%) cases in adults aged 18–64 years. Among children with COVID-19, 147 (estimated range = 5.7%–20%) were reported to be hospitalized, with 15 (0.58%–2.0%) admitted to an ICU (Figure 2). Among adults aged 18–64 years, the percentages of patients who were hospitalized (10%–33%), including those admitted to an ICU (1.4%–4.5%), were higher. Children aged <1 year accounted for the highest percentage (15%–62%) of hospitalization among pediatric patients with COVID-19. Among 95 children aged <1 year with known hospitalization status, 59 (62%) were hospitalized, including five who were admitted to an ICU. The percentage of patients hospitalized among those aged 1–17 years was lower (estimated range = 4.1%–14%), with little variation among age groups (Figure 2).

**FIGURE 2:**
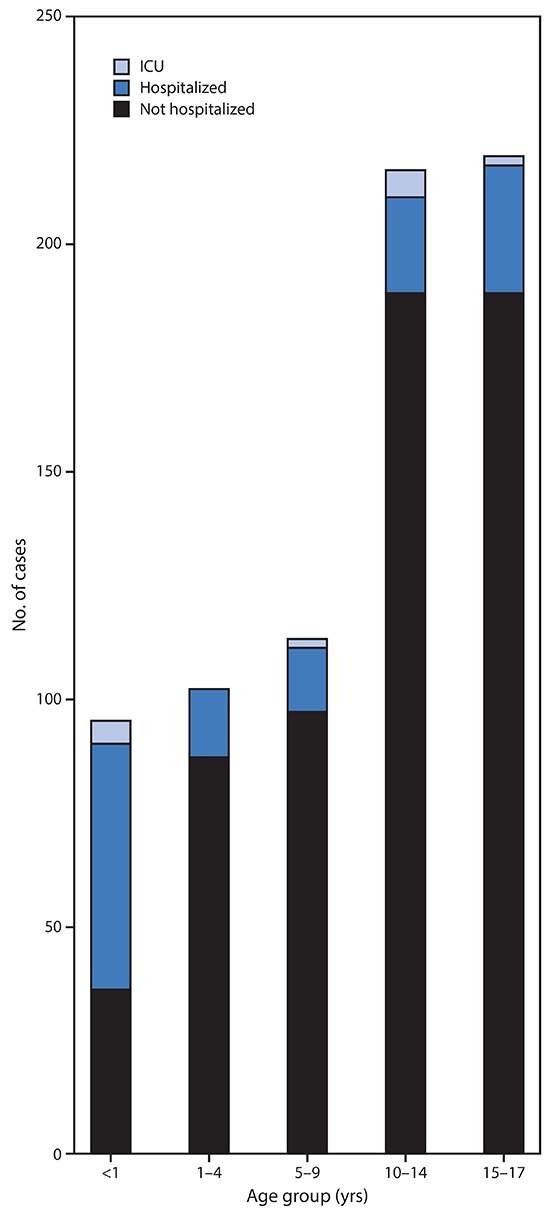
COVID-19 cases among children* aged <18 years, among those with known hospitalization status (N = 745),^†^ by age group and hospitalization status — United States, February 12–April 2, 2020 **Abbreviation:** ICU = intensive care unit. * Includes infants, children, and adolescents. ^†^ Number of children missing hospitalization status by age group: <1 year (303 of 398; 76%); 1–4 years (189 of 291; 65%); 5–9 years (275 of 388; 71%); 10–14 years (466 of 682; 68%); 15–17 years (594 of 813; 73%).

Among 345 pediatric cases with information on underlying conditions, 80 (23%) had at least one underlying condition. The most common underlying conditions were chronic lung disease (including asthma) (40), cardiovascular disease (25), and immunosuppression (10). Among the 295 pediatric cases for which information on both hospitalization status and underlying medical conditions was available, 28 of 37 (77%) hospitalized patients, including all six patients admitted to an ICU, had one or more underlying medical condition; among 258 patients who were not hospitalized, 30 (12%) patients had underlying conditions. Three deaths were reported among the pediatric cases included in this analysis; however, review of these cases is ongoing to confirm COVID-19 as the likely cause of death.

## Discussion

Among 149,082 U.S. cases of COVID-19 reported as of April 2, 2020, for which age was known, 2,572 (1.7%) occurred in patients aged <18 years. In comparison, persons aged <18 years account for 22% of the U.S. population (*3*). Although infants <1 year accounted for 15% of pediatric COVID-19 cases, they remain underrepresented among COVID-19 cases in patients of all ages (393 of 149,082; 0.27%) compared with the percentage of the U.S. population aged <1 year (1.2%) (*3*). Relatively few pediatric COVID-19 cases were hospitalized (5.7%–20%; including 0.58%–2.0% admitted to an ICU), consistent with previous reports that COVID-19 illness often might have a mild course among younger patients (*4*,*5*). Hospitalization was most common among pediatric patients aged <1 year and those with underlying conditions. In addition, 73% of children for whom symptom information was known reported the characteristic COVID-19 signs and symptoms of fever, cough, or shortness of breath.

These findings are largely consistent with a report on pediatric COVID-19 patients aged <16 years in China, which found that only 41.5% of pediatric patients had fever, 48.5% had cough, and 1.8% were admitted to an ICU (*4*). A second report suggested that although pediatric COVID-19 patients infrequently have severe outcomes, the infection might be more severe among infants (*5*). In the current analysis, 59 of 147 pediatric hospitalizations, including five of 15 pediatric ICU admissions, were among children aged <1 year; however, most reported U.S. cases in infants had unknown hospitalization status.

In this preliminary analysis of U.S. pediatric COVID-19 cases, a majority (57%) of patients were males. Several studies have reported a majority of COVID-19 cases among males (*4*,*9*), and an analysis of 44,000 COVID-19 cases in patients of all ages in China reported a higher case-fatality rate among men than among women (*10*). However, the same report, as well as a separate analysis of 2,143 pediatric COVID-19 cases from China, detected no substantial difference in the number of cases among males and females (*5*,*10*). Reasons for any potential difference in COVID-19 incidence or severity between males and females are unknown. In the present analysis, the predominance of males in all pediatric age groups, including patients aged <1 year, suggests that biologic factors might play a role in any differences in COVID-19 susceptibility by sex.

The findings in this report are subject to at least four limitations. First, because of the high workload associated with COVID-19 response activities on local, state, and territorial public health personnel, a majority of pediatric cases were missing data on disease symptoms, severity, or underlying conditions. Data for many variables are unlikely to be missing at random, and as such, these results must be interpreted with caution. Because of the high percentage of missing data, statistical comparisons could not be conducted. Second, because many cases occurred only days before publication of this report, the outcome for many patients is unknown, and this analysis might underestimate severity of disease or symptoms that manifested later in the course of illness. Third, COVID-19 testing practices differ across jurisdictions and might also differ across age groups. In many areas, prioritization of testing for severely ill patients likely occurs, which would result in overestimation of the percentage of patients with COVID-19 infection who are hospitalized (including those treated in an ICU) among all age groups. Finally, this analysis compares clinical characteristics of pediatric cases (persons aged <18 years) with those of cases among adults aged 18–64 years. Severe COVID-19 disease appears to be more common among adults at the high end of this age range (*6*), and therefore cases in young adults might be more similar to those among children than suggested by the current analysis.

As the number of COVID-19 cases continues to increase in many parts of the United States, it will be important to adapt COVID-19 surveillance strategies to maintain collection of critical case information without overburdening jurisdiction health departments. National surveillance will increasingly be complemented by focused surveillance systems collecting comprehensive case information on a subset of cases across various health care settings. These systems will provide detailed information on the evolving COVID-19 incidence and risk factors for infection and severe disease. More systematic and detailed collection of underlying condition data among pediatric patients would be helpful to understand which children might be at highest risk for severe COVID-19 illness.

This preliminary examination of characteristics of COVID-19 disease among children in the United States suggests that children do not always have fever or cough as reported signs and symptoms. Although most cases reported among children to date have not been severe, clinicians should maintain a high index of suspicion for COVID-19 infection in children and monitor for progression of illness, particularly among infants and children with underlying conditions. However, these findings must be interpreted with caution because of the high percentage of cases missing data on important characteristics. Because persons with asymptomatic and mild disease, including children, are likely playing a role in transmission and spread of COVID-19 in the community, social distancing and everyday preventive behaviors are recommended for persons of all ages to slow the spread of the virus, protect the health care system from being overloaded, and protect older adults and persons of any age with serious underlying medical conditions. Recommendations for reducing the spread of COVID-19 by staying at home and practicing strategies such as respiratory hygiene, wearing cloth face coverings when around others, and others are available on CDC’s coronavirus website at https://www.cdc.gov/coronavirus/2019-ncov/prevent-getting-sick/prevention.html.

**Summary**

**What is already known about this topic?**

Data from China suggest that pediatric coronavirus disease 2019 (COVID-19) cases might be less severe than cases in adults and that children (persons aged <18 years) might experience different symptoms than adults.

**What is added by this report?**

In this preliminary description of pediatric U.S. COVID-19 cases, relatively few children with COVID-19 are hospitalized, and fewer children than adults experience fever, cough, or shortness of breath. Severe outcomes have been reported in children, including three deaths.

**What are the implications for public health practice?**

Pediatric COVID-19 patients might not have fever or cough. Social distancing and everyday preventive behaviors remain important for all age groups because patients with less serious illness and those without symptoms likely play an important role in disease transmission.

## Footnotes

* https://www.cdc.gov/coronavirus/2019-ncov/downloads/pui-form.pdf.
